# Which radiological parameters of the coracoid process influence the diagnosis of atraumatic subscapularis tears? Systematic review and meta-analysis

**DOI:** 10.1007/s00590-025-04475-2

**Published:** 2025-09-06

**Authors:** Rubén Dario Arias Pérez, Alejandro Jaramillo Quiceno, Alejandro Mejia Bustamante

**Affiliations:** 1 Pontifical Bolivarian University, Medellín, Colombia; 2 Salud Sura, Medellín, Colombia

**Keywords:** Rotator Cuffs, Subscapularis, Coracoid Process, Coracohumeral Impingement Syndrome

## Abstract

**Introduction:**

Accurate diagnosis of subscapularis tears remains challenging due to the limitations of physical examinations and imaging techniques. Therefore, specific radiological parameters have been proposed as predictors of atraumatic subscapularis tears to improve diagnostic sensitivity and accuracy. These parameters include coracohumeral distance (CHD), coracoglenoid angle (CGA), coracoid angle (CA), coracoid overlap (CO), and coracohumeral angle (CHA). However, well-accepted cutoff values are still lacking, and there is no consensus on its clinical usefulness.

**Materials and methods:**

The PubMed, Scopus, and Cochrane Library databases were queried in July 2024. Inclusion criteria focused on studies that reported MRI-based radiological measurements of the coracoid process in patients with subscapularis tears versus controls. A meta-analysis was performed to evaluate outcomes, with data reported as raw mean difference (MD) and 95% confidence interval (CI).

**Results:**

Fourteen studies involving 1,692 patients with subscapularis tears and 1,648 controls were included. Significant findings include a smaller axial CHD in the subscapularis tear group compared to controls (MD, − 1.67; 95% CI, − 2.61 to − 0.72; *P* = 0.002). The sagittal CHD was also reduced in the tear group (MD, − 1.43; 95% CI, − 1.89 to − 0.98; *P* < 0.0001). The CGA was (MD, − 1.15; 95% CI, − 2.20 to − 0.10; *P* = 0.032), and the CA was also reduced (MD, − 18.63; 95% CI, − 35.60 to − 1.66; *P* = 0.042). The CO showed no significant difference between the tear and control groups (MD, 1.68; 95% CI, − 1.27 to 4.62; *P* = 0.21). In contrast, the CHA was increased in the tear group (MD, 3.71; 95% CI, 2.32–5.11; *P* < 0.01).

**Conclusion:**

Several radiological parameters, including CA, CHA, CGA, and axial and sagittal CHD, demonstrated statistically significant differences between patients with and without atraumatic subscapularis tears. Among them, CHA appears to be the most promising due to its consistent association and low heterogeneity. However, substantial variability across studies and limited data for certain parameters underscore the need for further prospective research to validate their diagnostic value and establish standardized imaging protocols.

**Study Design:**

Systematic review and meta-analysis; Level of evidence, III.

## Introduction

The subscapularis tendon is the largest of the rotator cuff, and it has been recognized as a crucial component in shoulder function and pathology [1, 2]. Unlike the supraspinatus, isolated subscapularis tears are infrequent and mostly associated with degenerative changes in the tendon. Recent advancements in evaluation methods, such as magnetic resonance imaging (MRI) and arthroscopy, have increased our understanding and interest in these lesions [3]. Several studies report that the prevalence of subscapularis tears is higher than previously believed, with findings by et al. suggesting prevalence rates up to 10.1% [4]. This has led to increased publications reaching a 12–50% prevalence in patients undergoing arthroscopy [3].

Although MRI has good sensitivity for diagnosing supraspinatus and infraspinatus injuries, accuracy for detecting subscapularis tears ranges from 56 to 67.6%, and this varies depending on the magnetic field of the MRI [5–7]. Furthermore, the accuracy of MRI in diagnosing subscapularis tears is influenced by the thickness and size of the lesion [7]. Injuries occurring in the upper third of the tendon insertion, which is notably difficult to visualize with MRI, account for most subscapularis tears [8]. Consequently, diagnosing subscapularis tendon lesions remains difficult due to the low sensitivity of both physical examination findings and imaging modalities [3, 9].

Degenerative tears of the subscapularis tendon are a well-recognized source of shoulder pain and dysfunction, yet they have historically been underestimated and underdiagnosed [3]. Recent research has increasingly focused on the etiological factors contributing to subscapularis tears, underscoring the critical role of this tendon in shoulder pathology. Subcoracoid impingement has emerged as a significant contributor to degenerative subscapularis [10]. The pathological mechanism involves a structural narrowing in the subcoracoid space. This degeneration can result from repeated friction between the rotator cuff and the bone. However, our understanding of the detailed mechanisms behind these lesions is still incomplete, indicating that further research is needed [11].

Clinical practice typically assesses the severity of tendon injuries on MRI by observing changes in tendon morphology and signal, but this approach has limitations [12, 13]. Recent studies have suggested that subcoracoid impingement and coracoid morphological variation may play a critical role in subscapularis tear, and indirect imaging signs and radiological parameters might be associated with the diagnosis of tendon tear [3]. Identifying reliable predictors for subscapularis tendon tears could improve diagnostic sensitivity and accuracy [14].

**Fig. 1:**
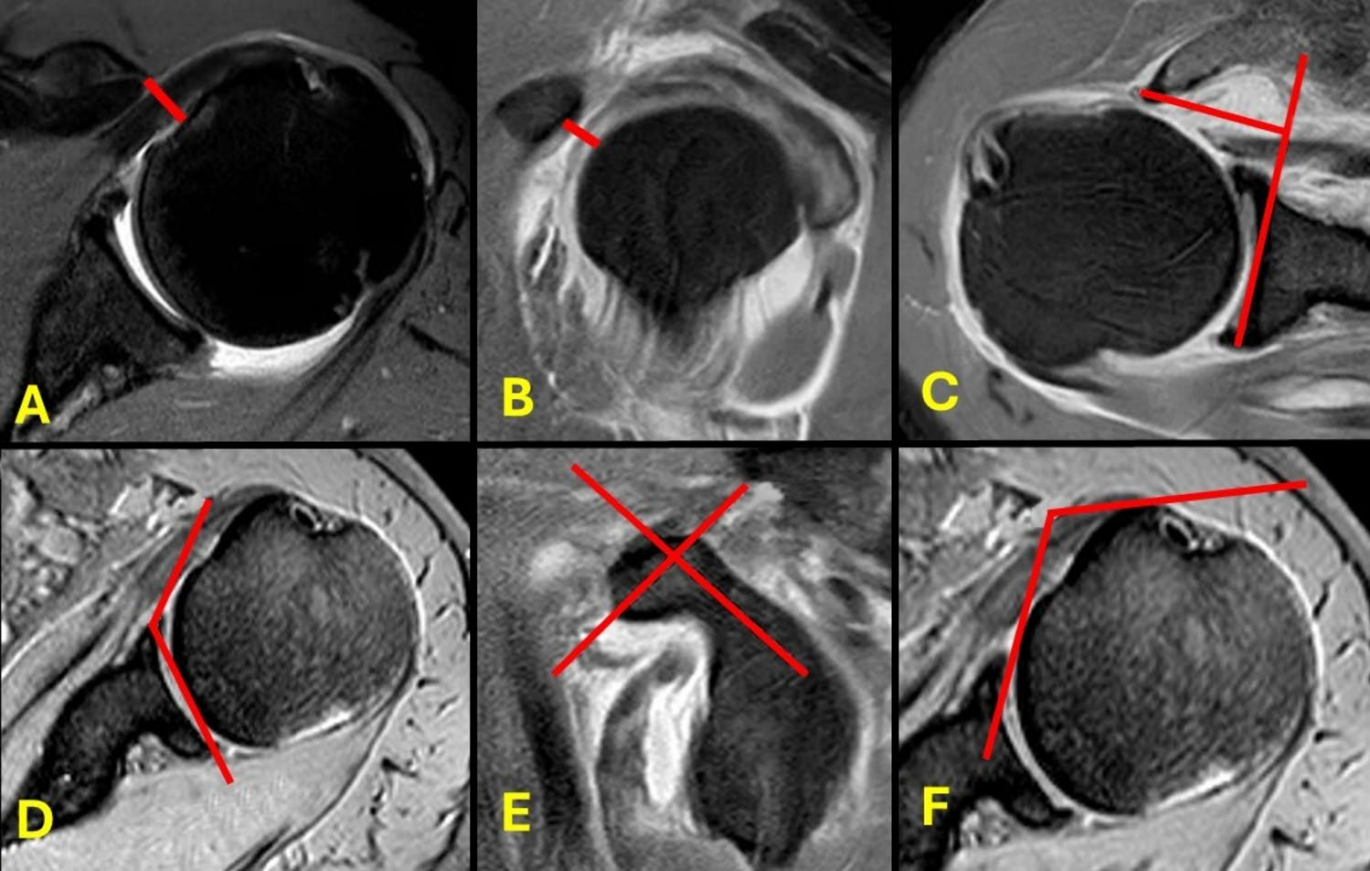
Most frequently reported radiological parameters of the coracoid. **A** Axial coracohumeral distance. **B** Sagittal coracohumeral distance. **C** Coracoid overlap. **D** Coracoglenoid angle. **E** Coracoid angle. **F** Coracohumeral angle

Among the radiological parameters used to increase suspicion of subscapularis tear, the following are especially significant: the coracohumeral distance (CHD), the shortest distance between the coracoid process and the lesser tuberosity on axial and sagittal images [15]; the coracoid angle (CA), the angle between the long axes of the proximal and distal segments of the coracoid process on sagittal images [12]; the coracoglenoid angle (CGA), the angle between a line from the most distal part of the coracoid process to the anterior glenoid corner and a line along the glenoid surface on axial images [12]; the coracoid overlap (CO), the distance from the most distal part of the coracoid process to the glenoid on axial images [16]; and the coracohumeral angle (CHA), the angle between a line drawn from the inner surface of the coracoid process to the lateral surface of humeral head and a line from the inner surface of the coracoid process to the medial surface of the humeral head on axial images [17] (Fig. 1). In addition, other less reported parameters have been described including the coracohumeral index, glenoid version, coracoid total length, coracoid tip-glenoid angle, coraco-lesser tuberosity angle, lesser tuberosity angle, and lesser tuberosity height [11, 18–20]. However, well-accepted cutoff values are still lacking, and there is no consensus on which parameters are the most reliable [3, 21].

### Objectives

This systematic review and meta-analysis aimed to identify the most valuable coracoid-related radiological parameters associated with the diagnosis of subscapularis tears. It was hypothesized that patients with subscapularis tears exhibit distinct differences in certain radiological measurements of the coracoid process compared to those without subscapularis tears.

## Materials and methods

### Search strategy

This systematic review and meta-analysis adhered to the Preferred Reporting Items for systematic reviews and meta-analyses (PRISMA) 2020 statement [22]. A systematic and comprehensive online literature search was conducted using PubMed, Scopus, and Cochrane Library from database inception to July 1, 2024. There were no restrictions on the publication type, journal, and language. The search terms were: (Coracoid OR coracoidal OR coracoids OR coracohumeral) AND (subscapularis tear).

### Eligibility criteria

The inclusion criteria for this review were as follows: (1) patients in the experimental group with subscapularis tears confirmed by MRI and patients in the control group without subscapularis tears confirmed by MRI, (2) studies reporting radiological findings related to the coracoid process that were measured on MRI with the arm in a neutral position, (3) evidence levels of 1 to 4, and (4) studies published in English or Spanish. The exclusion criteria were as follows: (1) studies with the duplication of patients; (2) cadaveric, biomechanical, or animal studies; (3) studies that do not report complete quantitative data; (4) nonclinical studies, systematic reviews, meta-analyses, case reports, surgical technique, basic science articles, commentaries, letters, expert opinions, and revisions.

### Study selection

All the publications found by the search terms were reviewed. An initial literature search was conducted, and studies with identical patient populations or duplicates were assessed and removed. Manual searches were also performed for articles the electronic search could have missed. Abstracts were independently screened for potential inclusion by two reviewers. Any discrepancies were resolved through discussion with a third author; after this initial screening, relevant studies were read in full to assess whether they met the pre-established eligibility criteria.

### Data collection and extraction

The following data were extracted from the full-text version of the included articles: (1) Study characteristics: lead author name, publication date, publication journal, study design, study period, country, and level of evidence; (2) patient characteristics: age, sex, diagnostic method used, and blinding procedures; and (3) result of radiological measurements. If results were reported according to subscapularis tear type, only data regarding full-thickness or complete tears were considered. Data were collected as mean and standard deviation (SD) for continuous outcomes and frequency and percentage for categorical data. When mean and SD were unavailable, the data were transformed into means and SD [23, 24]. Two reviewers independently extracted the data. Any discrepancies were resolved through discussion with a third author.

### Risk of bias assessment

The methodological quality of each study was assessed according to the Newcastle–Ottawa Scale (NOS). The scale was assessed across three key domains: study population selection, group comparability, and measurement of exposure factors. The maximum possible score is 9, with 0–3 indicating low quality, 4–6 representing medium quality, and 7–9 signifying high quality. The scale was assessed by two independent reviewers, and the average score was used as the final score.

### Statistical analysis

Continuous variables were expressed as means with SD, while categorical variables were reported as frequencies and percentage. The primary analysis used the raw mean difference as the outcome measure, accompanied by 95% confidence intervals (CI) to assess the precision of the estimates. A random effects model was employed, incorporating the restricted maximum likelihood estimator along with the Knapp-Hartung adjustment. A fixed effects model was applied when heterogeneity was low, defined as *I*^2^ < 50% or tau^2^ approaching zero. Heterogeneity was evaluated using both the tau^2^ estimate and the *I*^2^ statistic. Statistical significance was determined with a threshold of *P* < 0.05. All meta-analyses were performed using the open-source software RStudio, specifically employing the metafor package for analysis and visualization.

## Results

### Identification of studies

**Fig. 2:**
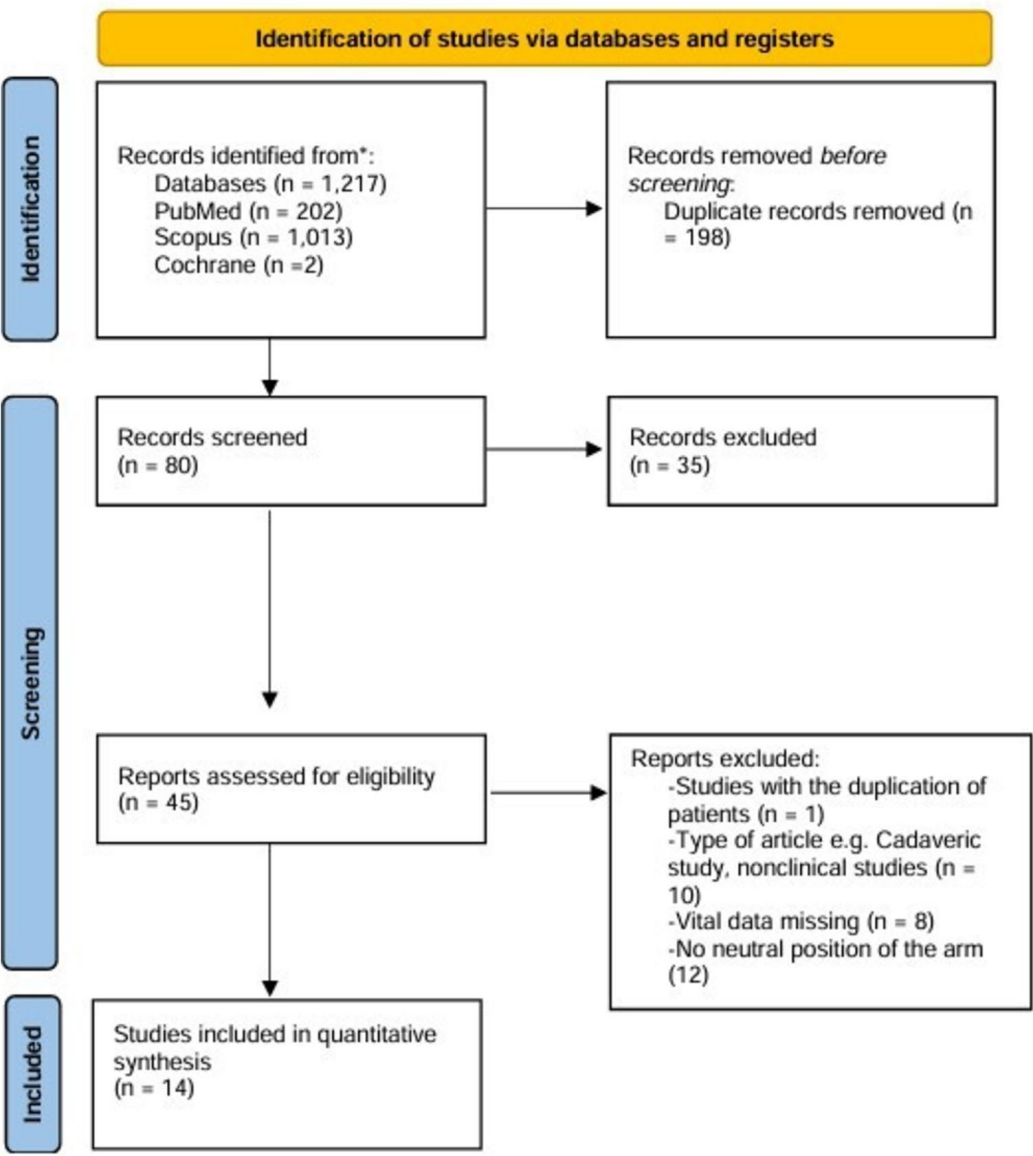
PRISMA (Preferred Reporting Items for Systematic Reviews and Meta-analyses) 2020 flowchart showing the selection process of studies

The electronic search yielded a total of 1,217 records (PubMed: *n* = 202; Scopus: *n* = 1,013; Cochrane Library: *n* = 2). After removing duplicates, 80 studies were screened based on titles and abstracts. Subsequently, 45 full studies were read to assess their eligibility criteria and compliance. This process led to the inclusion of 14 studies in this systematic review. The PRISMA flow diagram is presented in Fig. 2.

### Characteristics of included studies and patients

Fourteen studies were published between 2016 and 2024 [11, 16–18, 25–34]. Only one study was published in Spanish [33], and the rest of the articles were published in English. Most studies have a level of evidence grade III. In nine of the articles, arthroscopy and MRI were used to determine the presence or absence of subscapularis tear. No studies were at high risk of reporting bias. Fourteen studies included a total of 1,692 patients with subscapularis tears in the experimental group and 1,648 patients without subscapularis tears in the control group. The mean age of patients in the experimental group was 57.6 years, while the mean age of those in the control group was 52.3 years. In the experimental group, 50.6% of the patients were men and 49.4% were women. In contrast, in the control group without subscapularis tears, 46% were men and 54% were women. The characteristics of the included studies and patients are shown in Table 1.

**Table 1:** Characteristics of included studies and patients

Lead author	Year	Journal	Study period	Country	Experimental group (n)	Control group (n)	Men/Women experimental	Men/Women control	Mean age experimental (y ± SD)	Mean age control (y ± SD)	Method of diagnosis	Study design	Level of evidence	NOS Score
Cardenas	2019	Rev Chil Ortop Traumatol	NR	Chile	46	36	NR	NR	51.7 ± 6.8	50.1 ± 6.1	MRI	Case–control design	III	7
Cetinkaya	2016	Arthroscopy	2004–2015	Turkey	141	78	48/93	22/56	57.85 ± 10.44	55.46 ± 11.73	Arthroscopy and MRI	Case–control design	III	8
Cetinkaya	2023	Turk J Med Sci	2016–2018	Turkey	16	16	6/10	6/10	49.8 ± 8.7	49.8 ± 8.7	Arthroscopy and MRI	Case–control design	III	7
Hay-Man	2016	J Orthop Trauma Rehabil	2011	China	13	120	6/7	57/59	58.8 ± 17.5	51.6 ± 13.8	MRI	Retrospective cohort	III	7
Ilyas G	2023	Med Sci Monit	2019–2023	Turkey	69	118	28/41	51/67	55.7 ± 11.1	53.3 ± 13.8	Arthroscopy and MRI	Case–control design	III	8
JB Seo	2020	Orthop Traumatol Surg Res	2016–2018	Korea	114	57	75/39	41/16	60.30 ± 10.7	51.3 ± 9.8	Arthroscopy and MRI	Case–control design	IV	7
Kilic AI	2024	Arthroscopy	2011–2021	USA	328	249	NR	127/122	61.8 ± 10.1	59.4 ± 10.1	Arthroscopy and MRI	Case–control design	III	8
Kucukciloglu	2022	Clin Orthop Surg	2018–2020	Cyprus	85	213	62/23	129/84	38.08 ± 9.08	33.02 ± 10.14	MRI	Retrospective cohort	III	8
Leite	2019	J Shoulder Elbow Surg	2009–2018	Portugal	36	156	NR	NR	NR	NR	MRI	Case–control design	IV	7
Leite	2020	J Shoulder Elbow Surg	2009–2019	Portugal	188	98	NR	NR	NR	NR	MRI	Case–control design	III	7
Mi Y	2024	Jt Dis Relat Surg	2018–2022	China	60	60	29/31	29/31	58.4 ± 8.4	46.8 ± 11.5	MRI	Case–control design	III	8
Siriwanarangsun	2023	Quant Imaging Med Surg	2018–2019	Thailand	29	47	11/18	24/23	65 ± 11	58 ± 18	Arthroscopy and MRI	Case–control design	III	8
Xu W (Training cohort)	2022	BMC Musculoskelet Disord	2017–2020	China	184	276	58/126	92/184	62.55 ± 9.03	59.76 ± 9.42	Arthroscopy and MRI	Case–control design	III	8
Xu W (Validation cohort)	2022	BMC Musculoskelet Disord	2016–2021	China	86	114	27/59	39/75	63.12 ± 8.82	60.48 ± 9.13	Arthroscopy and MRI	Case–control design	III	8
Yoon SH	2020	J Orthop	2016–2019	Korea	297	57	200/97	41/16	57.8 ± 8.4	51.3 ± 9.8	Arthroscopy and MRI	Case–control design	IV	7

NR: No reported

### Radiological measurements

The axial CHD, sagittal CHD, CO, CGA, and CA were the most frequently reported measurements. The CHA only was reported by Siriwanarangsun et al.[30] and Ilyas G et al.[17]. Other measures related to subscapularis tear were reported in a few studies and were not included in the meta-analysis. These measures include that the coraco-lesser tuberosity angle was reported only by JB Seo et al.[11]. The coraco-coracoid base angle and coracoglenoid distance that were reported only by Cetinkaya et al.[25]. The coracoid body-glenoid angle, coracoid tip-glenoid angle, coracoid tip-body angle, and the coraco-scapular angle were reported by Ilyas et al.[17]. The coracoid proximal length, coracoid distal length, coracoid total length, and the coracoid length ratio were reported by Leite et al.[18]. The coracoid-glenoid tubercle distance was reported by Kucukciloglu et al.[28]. The morphological classification of the coracoid was also not included due to the variability and lack of consensus on this classification in the literature [18, 30].

### Axial coracohumeral distance

**Fig. 3:**
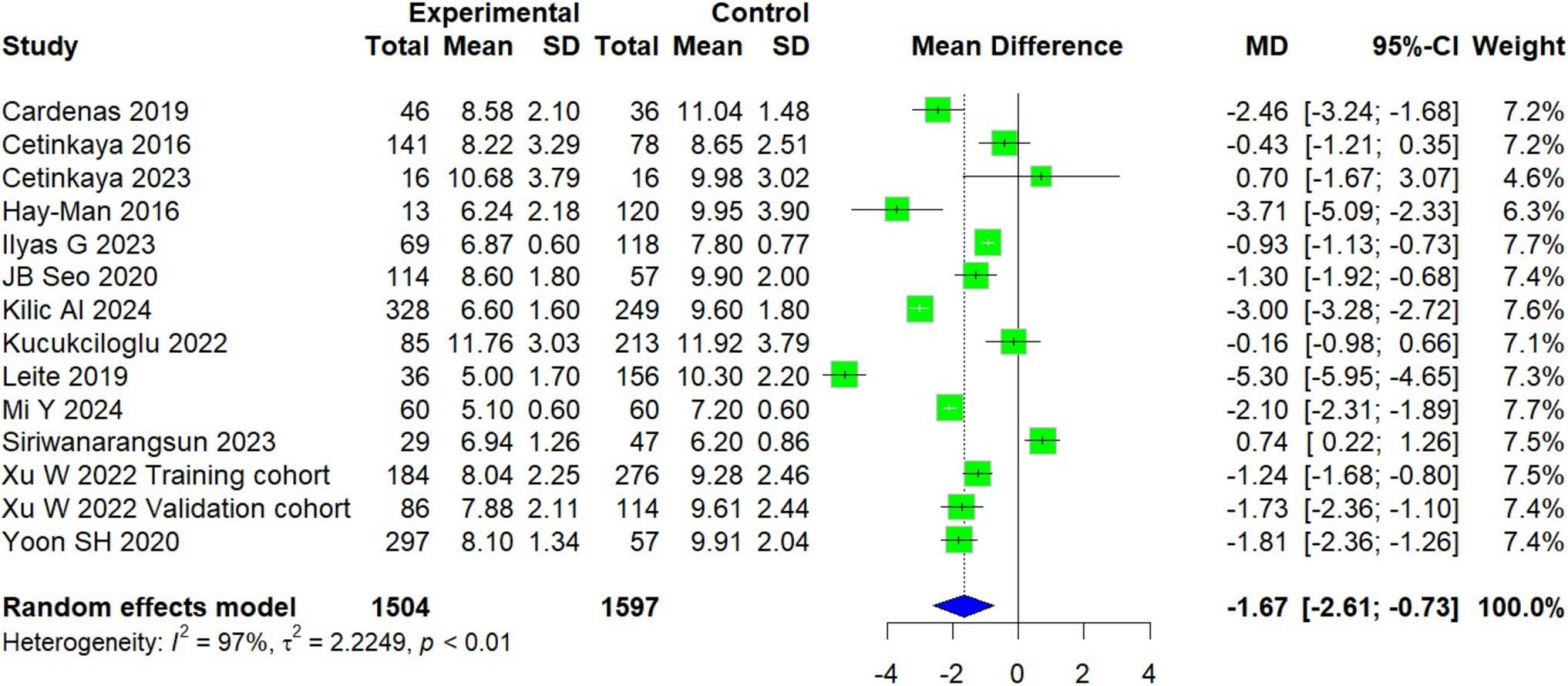
Forest plot of the axial coracohumeral distance. Data were pooled with a random effects model

A total of 3,101 patients were included across 13 studies conducted in various countries, including Turkey, the USA, Portugal, Korea, China, and others [11, 16, 17, 25–34]. The experimental group, consisting of 1,504 patients with subscapularis tears, was compared to a control group of 1,597 patients. On average, the experimental group showed a measurement that was 1.67 mm smaller than that of the control group [95% CI, − 2.61 to − 0.73; *P* = 0.002]. The analysis indicated high heterogeneity (*I*^2^ = 97%, *P* < 0.01) (Fig. 3).

### Sagittal coracohumeral distance

**Fig. 4:**
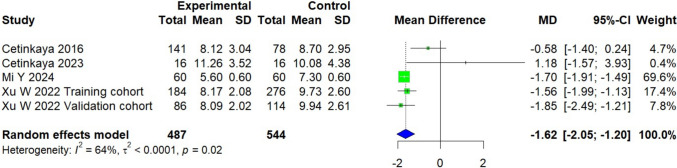
Forest plot of the sagittal coracohumeral distance. Data were pooled with a random effects model

A total of 1,031 patients were included across four studies, with two conducted in China and two in Turkey [25, 26, 31, 34]. The experimental group, comprising 487 patients with subscapularis tears, was compared to a control group of 544 patients. On average, the experimental group exhibited a measurement that was 1.62 mm smaller than that of the control group [95% CI, − 2.05 to − 1.20; *P* < 0.0001]. The analysis revealed moderate heterogeneity (*I*^2^ = 64%, *P* = 0.02) (Fig. 4).

### Coracoid overlap

**Fig. 5:**
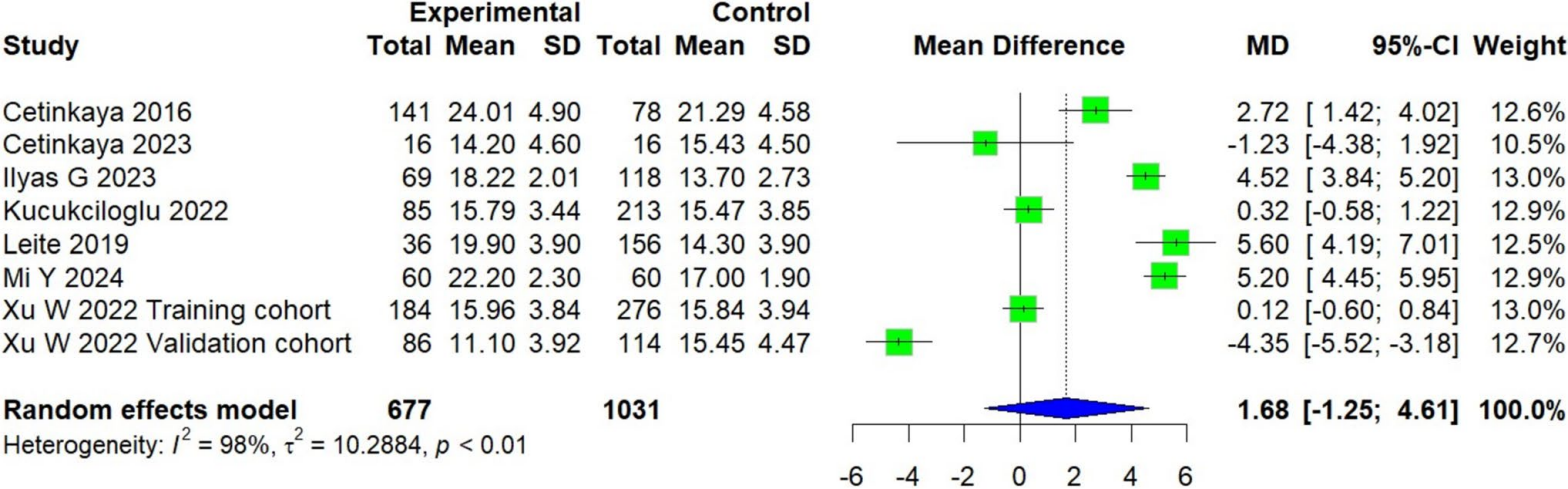
Forest plot of the coracoid overlap. Data were pooled with a random effects model

A total of 1,714 patients were included across seven studies, six of which were conducted in Asian countries [16, 17, 25, 26, 28, 31, 34]. The experimental group, consisting of 677 patients with subscapularis tears, was compared to a control group of 1,031 patients. The experimental group presented a mean difference 1.68 mm higher than that of the control group [95% CI, − 1.27 to 4.61; *P* = 0.21]. A high level of heterogeneity was observed (*I*^2^ = 98%, *P* < 0.01) (Fig. 5).

### Coracoglenoid angle

**Fig. 6:**
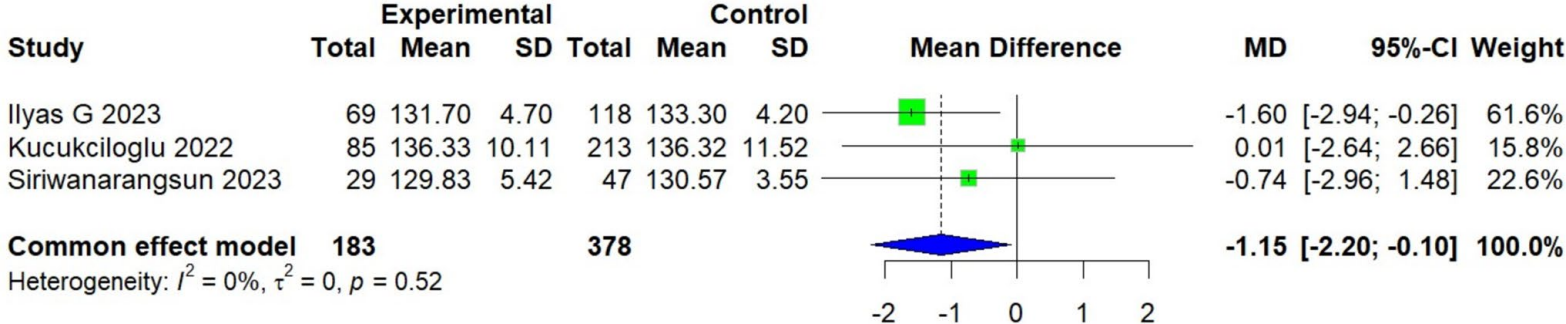
Forest plot of the coracoglenoid angle. Data were pooled with a fixed effects model

A total of 561 patients were included in three studies, all conducted in Asian countries [17, 28, 30]. The experimental group, consisting of 183 patients with subscapularis tears, was compared to a control group of 378 patients. On average, the experimental group demonstrated a measurement that was 1.15 grades smaller than the control group [95% CI, − 2.20 to − 0.10; *P* = 0.032]. The analysis revealed low heterogeneity (*I*^2^ = 0%, *P* = 0.52) (Fig. 6).

### Coracoid angle

**Fig. 7:**
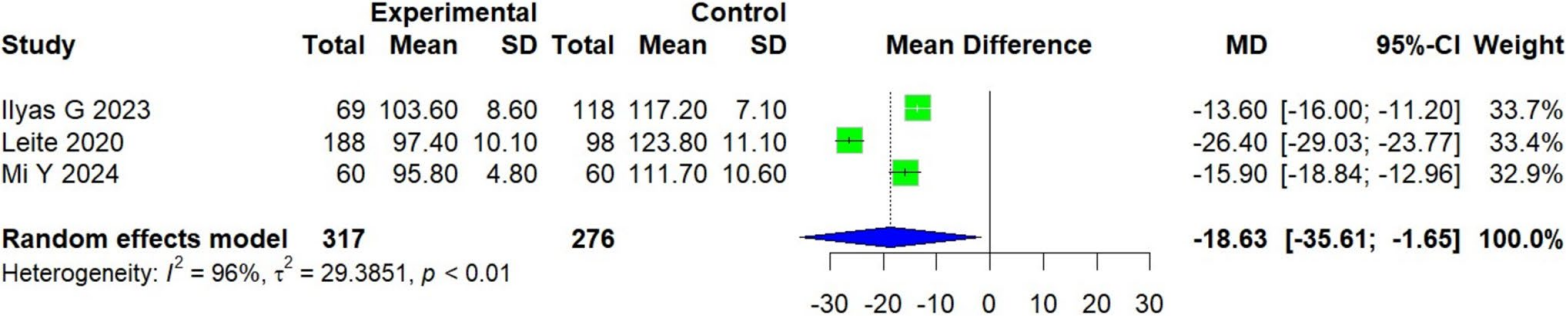
Forest plot of the coracoid angle. Data were pooled with a random effects model

A total of 593 patients were included across three studies conducted in Turkey, Portugal, and China [17, 18, 31]. The experimental group, consisting of 317 patients with subscapularis tears, was compared to a control group of 276 patients. On average, the experimental group exhibited a measurement that was 18.63 grades smaller than that of the control group [95% CI, − 35.60 to − 1.65; *P* = 0.042]. A high level of heterogeneity was observed (*I*^2^ = 96%, *P* < 0.01) (Fig. 7).

### Coracohumeral angle

**Fig. 8:**
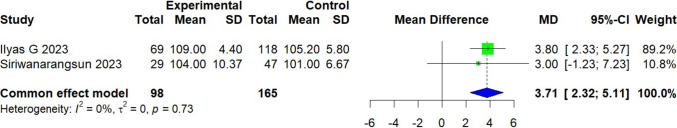
Forest plot of the coracohumeral angle. Data were pooled with a fixed effects model

A total of 263 patients were included in two studies conducted in Turkey and Thailand [17, 30]. The experimental group, consisting of 98 patients with subscapularis tears, was compared to a control group of 165 patients. On average, the experimental group exhibited a measurement that was 3.71 grades higher than that of the control group [95% CI, 2.32 to 5.11; *P* < 0.01]. A low level of heterogeneity was observed (*I*^2^ = 0%, *P* = 0.73) (Fig. 8).

## Discussion

This systematic review and meta-analysis aimed to determine whether specific coracoid-related radiological parameters are associated with atraumatic subscapularis tendon tears. The main findings suggest that certain measurements, particularly the coracohumeral angle (CHA), coracoglenoid angle (CGA), coracoid angle (CA), and both axial and sagittal coracohumeral distances (CHD), demonstrate statistically significant differences between patients with and without subscapularis tears. However, not all parameters presented consistent results across studies, and substantial heterogeneity was noted, particularly for axial CHD and CA.

Among all parameters, the CHA stands out as particularly promising. It demonstrated a mean difference (MD) of 3.71° between groups, with minimal heterogeneity and consistent results across the two studies that reported this measurement [17, 30]. Previous research supports these findings: Yu JF et al.[35] and Asal N et al.[12] have reported an increase in the CHA in patients with subscapularis injuries compared to controls. Moreover, Ilyas G et al.[17] have indicated that with a cutoff value of 107.25° of the CHA, the sensitivity and specificity to detect subscapularis tear were 67% and 68%, respectively. Although evidence is currently limited to a small number of studies, the reproducibility, statistical significance, and potential clinical relevance of CHA suggest it may serve as a useful radiological marker. However, further prospective studies are needed to validate its diagnostic performance and determine its integration into clinical protocols.

Similarly, the sagittal CHD, though reported in only four studies [25, 26, 31, 34], also showed a statistically significant MD of − 1.62 mm with moderate heterogeneity (*I*^2^ = 64%), reinforcing its potential clinical value. Despite being less frequently reported than axial CHD, sagittal CHD appears to be a promising radiological marker for the detection of subscapularis tears. Some authors have even suggested that it may outperform axial CHD in diagnostic accuracy for these lesions [36]. Its consistent association with tendon pathology across studies supports its potential clinical relevance, warranting further investigation in standardized imaging protocols.

In contrast, the axial CHD, one of the most widely reported variables, exhibited significant heterogeneity (*I*^2^ = 97%). Although our pooled analysis confirmed a significant reduction in the tear group (MD, − 1.67 mm; *P* = 0.002), the high variability in cutoff values across studies suggests a lack of standardization in measurement protocols. For example, Leite et al. [16] reported a CHD threshold of 7.6 mm with a sensitivity of 84.4% and specificity of 88.6%, while Reichel et al. [37] suggested a value < 9.5 mm yielded sensitivity and specificity of 83.6% and 83.9%, respectively. These discrepancies could stem from differences in MRI slice thickness, patient positioning, and anatomical interpretation of the landmarks used to measure CHD.

The CA also showed a statistically significant MD of − 18.63° between groups [17, 18, 31]. However, the heterogeneity was substantial (*I*^2^ = 96%). Variations in anatomical reference points and imaging orientation may partly explain these inconsistencies. Leite et al. [18] were among the first to propose the CA as a potential diagnostic marker for subscapularis tears. Our findings support this association, as patients with subscapularis pathology consistently exhibited smaller CA values than controls, with reductions ranging from approximately 13.6° to 26.4° across studies. Nevertheless, the wide confidence intervals and significant between-study heterogeneity currently undermine the parameter’s clinical reliability, highlighting the need for further standardization and validation.

In contrast, CO, despite being one of the most frequently used measures in clinical practice [16, 17, 25, 26, 28, 31, 34], did not show a significant difference between groups (*P* = 0.21) and presented high heterogeneity (*I*^2^ = 98%). These finding questions the isolated utility of CO in diagnosing subscapularis pathology, especially in the absence of standardized cutoff values. Reported thresholds vary widely: Cetinkaya et al. [25] suggested a cutoff of 22.85 mm, yielding a sensitivity of 62% and specificity of 64%, while Ilyas et al. [17] proposed a value of 16.5 mm with an improved sensitivity and specificity of 83% and 80%, respectively. This variability reflects the methodological differences across studies and highlights the limitations of CO as a reproducible diagnostic tool. Although some individual studies reported significant associations, the overall pooled analysis does not support CO as a reliable or consistent parameter for identifying subscapularis pathology (Fig. 5).

The CGA, though less frequently studied, showed a consistent and statistically significant MD of − 1.15° with low heterogeneity [17, 28, 30]. While the absolute difference appears small, its reproducibility may hold diagnostic value, particularly when used in combination with other imaging parameters. Likewise, some authors have reported lower CGA values in patients with subscapularis tears compared to healthy controls [13, 38]. Although the exact anatomical implications remain unclear, a decreased CGA may reflect subtle morphological variations in the anterior shoulder compartment that contribute to tendon pathology. Further studies are needed to clarify whether CGA reduction is a direct consequence of chronic anterior impingement or secondary to rotator cuff degeneration.

Anatomical variations in the coracoid process across different populations may partly explain the heterogeneity observed in radiological measurements. For instance, Fathi M. et al. reported significant ethnic differences (*P* < 0.05) in multiple coracoid dimensions, including length, base width, and tip thickness, between Indian, Chinese, and Myanmarese cadaveric and CT samples [38]. Consistent with these findings, CT-based morphometric studies have shown that Asian cohorts generally exhibit smaller coracoid and glenoid sizes compared to Caucasian and African populations, with statistically significant differences in almost all measured parameters [39].

Additionally, recent 3D-CT evaluations in Korean individuals confirmed that their coracoid processes are shorter than those reported in Western studies [40]. These consistent ethnogeographic differences in scapular morphology suggest that baseline anatomical variability might account for some of the inter-study discrepancies in CHD, CA, CHA, and other parameters. Therefore, without adjustments for population-specific reference values, direct comparisons across studies from Asia, Europe, or the Americas may inherently introduce bias and contribute to heterogeneity in meta-analysis results. Future multicenter and ethnically stratified studies are required to establish population-specific reference values and improve the diagnostic accuracy of these imaging metrics.

This study has several limitations that must be acknowledged. First, most included studies were retrospective in design and classified as level III evidence, which inherently limits the strength of causal inferences and introduces risks of selection and measurement bias. The absence of randomized controlled trials or prospective cohort designs reflects a gap in the current literature and restricts our ability to draw definitive diagnostic recommendations.

Second, considerable heterogeneity was observed across many of the evaluated parameters, particularly axial CHD, CA, and CO, which limits the generalizability of the pooled estimates. This variability likely results from methodological differences between studies, including patient selection criteria, MRI protocols (e.g., field strength, slice orientation), arm positioning, and inconsistent definitions of subscapularis tears. Although we attempted to mitigate this by including only studies that specified arm rotation during imaging, residual inconsistencies in measurement technique and anatomical landmark selection could have impacted the results.

Third, for certain parameters such as CHA and CGA, the number of available studies was limited, preventing robust subgroup analysis or exploration of population-specific factors. While we reported the geographic origin of the studies for contextual clarity, the review was not designed to investigate inter-population anatomical variability, and any such hypothesis remains speculative.

Despite these limitations, this study has important strengths. It is the most comprehensive synthesis to date examining coracoid-related radiological parameters in the context of atraumatic subscapularis tendon tears. A total of 14 studies and over 3,300 patients were included, providing a broad quantitative overview. By identifying CHA and sagittal CHD as parameters with statistically significant differences and low-to-moderate heterogeneity, the study offers valuable direction for future research focused on diagnostic imaging standardization and early detection of subscapularis pathology.

## Conclusion

Several radiological parameters, including CA, CHA, CGA, and both axial and sagittal CHD, showed statistically significant differences between patients with and without subscapularis tears. However, the clinical utility of most remains uncertain due to the substantial heterogeneity observed across studies and the lack of standardized imaging techniques or validated cutoff values. Axial CHD and CA demonstrated particularly high variability, while the magnitude of differences in CGA and sagittal CHD was limited, raising questions about their diagnostic relevance.

Among the evaluated parameters, the CHA appears most promising, supported by consistent statistical significance and minimal heterogeneity. Nevertheless, this conclusion is based on only two studies, and further validation is required before CHA can be incorporated reliably into diagnostic protocols.

In summary, while the current evidence highlights potential associations between specific coracoid-related measurements and subscapularis tears, it does not yet support definitive diagnostic use. Future prospective, multicenter studies with standardized imaging methodologies are needed to confirm the reliability and clinical applicability of these radiological parameters.

## Data Availability

No datasets were generated or analysed during the current study.
